# Interleukin 10 Attenuates Angiotensin II-Induced Aortic Remodelling by Inhibiting Oxidative Stress-Induced Activation of the Vascular p38 and NF-*κ*B Pathways

**DOI:** 10.1155/2022/8244497

**Published:** 2022-04-26

**Authors:** Ming Qiu, Huanyu Shu, Lu Li, Yejiao Shen, Yunfan Tian, Yue Ji, Wei Sun, Yan Lu, Xiangqing Kong

**Affiliations:** ^1^School of Medicine, Southeast University, Nanjing, Jiangsu 210009, China; ^2^Department of Cardiology, The First Affiliated Hospital of Nanjing Medical University, Nanjing, Jiangsu 210029, China

## Abstract

Interleukin 10 (IL-10) is a probable anti-inflammatory factor that can attenuate hypertrophic remodelling caused by overloaded pressure and improve cardiac function. In this study, IL-10 was decreased in both the plasma of hypertensive patients and the aortic vessels of angiotensin II (Ang II)-induced hypertensive mice. IL-10 was unable to alter blood pressure in the case of Ang II-induced hypertension. The aortic thickness, collagen deposition, and the levels of fibrosis-associated markers, including collagen type I *α* 1 (Col1*α*1), connective tissue growth factor (CTGF), transforming growth factor-*β* (TGF-*β*), and matrix metalloproteinase 2 (MMP2), were significantly reduced in the IL-10 treatment group compared with the vehicle group after Ang II treatment. Moreover, IL-10 treatment significantly inhibited the number of CD45^+^ positive cells and the mRNA expression levels of proinflammatory cytokines in the vascular tissue of Ang II-infused mice. Furthermore, dihydroethidium (DHE) and 4hydroxynonenal (4-HNE) staining showed that IL-10 decreased Ang II-induced vascular oxidative stress and lipid peroxidation. Furthermore, IL-10 suppressed Ang II-induced proliferation, fibrosis, and inflammation of mouse vascular adventitial fibroblasts (mVAFs). Mechanistically, IL-10 suppressed the phosphorylation of p38 mitogen-activated protein (MAP) kinase and nuclear factor-*κ*B (NF-*κ*B) in Ang II-induced vascular fibrosis. In summary, our data indicated that IL-10, as a potential therapeutic target treatment, could limit the progression of Ang II-induced aortic remodelling.

## 1. Introduction

Hypertension is the key risk factor for cardiovascular disease, which has become the leading cause of death worldwide and accounts for more than 17.3 million deaths per year [1]. A long-term elevation of blood pressure promotes vascular remodelling, which can lead to vascular malfunction [2]. Fibrosis is often defined as a wound-healing response that has gone out of control [3], and a progressive fibrogenic process leads to further worsening of vascular stiffness and vasomotion [4]. In mild primary hypertensive patients, predominantly inward eutrophic remodelling is observed [5]. In hypertension, vascular fibrosis largely entails the deposition of extracellular matrix components, particularly collagen, in the arterial wall [6].

In contrast to acute inflammatory reactions, vascular fibrosis typically arises from chronic inflammatory reactions persisting for several months in which inflammation, damaged endothelial cells, and repair processes occur simultaneously. Growth factors, cytokines, and chemokines secreted by injured endothelial cells can stimulate the proliferation and recruitment of inflammatory cells across the provisional extracellular matrix (ECM) [3, 7, 8]. However, in hypertension, arteriole adventitia, an often neglected layer of the vascular wall, manifests an inflammatory phenotype with increased numbers of inflammatory cells [9]. Inflammation probably acts as a vital trigger for small and large vessel injury and remodelling in hypertension [10].

IL-10 has long been known as a potent anti-inflammatory factor that controls monocyte infiltration and deactivates various proinflammatory mediators [11, 12]. IL-10 treatment attenuates pressure overload–induced hypertrophic remodelling and improves heart function via signal transducer and activator of transcription 3 (STAT-3)-dependent inhibition of nuclear factor-*κ*B [13]. Previously, Raj Kishore showed that IL-10 inhibits inflammation and attenuates left ventricular remodelling after myocardial infarction via activation of STAT-3 [14].

Despite these observations, the role of IL-10 administration in the effect of vascular hypertrophy and remodelling in hypertension is not well understood. Meanwhile, the systemic therapeutic effect of IL-10 on vascular dysfunction in hypertension and the molecular mechanism remain to be studied.

Therefore, we hypothesize that the vascular remodelling process induced by hypertension can be ameliorated by recombinant IL-10 protein administration. Here, for the first time, we show that recombinant IL-10 therapy significantly attenuates vascular fibrosis, aortic cross-sectional area, and inflammatory cytokine gene expression in response to Ang II stimulation. Inflammation, vascular remodelling, and potential signalling mechanisms regulated by IL-10 were elucidated in this study.

## 2. Materials and Methods

### 2.1. Reagents and Antibodies

Angiotensin II (cat. no. A9535), Hanks' balanced salt solution (HBSS, cat. no. H1387), and type II collagenase (cat. no. C9722) were obtained from Sigma–Aldrich (Merck KGaA, Darmstadt, Germany). Osmotic minipumps (Cat no. Model 2002) were obtained from ALZET Corporation (Cupertino, CA, USA). Recombinant mouse IL-10 (cat. no. 417-ML-005) was purchased from R&D Systems, Inc. (MN, USA). Ten percent neutral formalin fix solution (cat. no. E672001) was purchased from Sangon Biotech (Shanghai, China). A human IL-10 ELISA kit (cat. no. E-EL-H6154) was purchased from Elabscience (Wuhan, China). Dulbecco's modified Eagle medium (DMEM, cat. no. 11965092), fetal bovine serum (FBS, cat. no. 16140071), penicillin–streptomycin (P/S, cat. no. 15070063), and EDTA-free trypsin (cat. no. 15050057) were purchased from Gibco (MA, USA). Polyvinylidene difluoride western blotting membranes (cat. no. 03010040001) were purchased from Roche (Basel, Switzerland). ChromatoPur bovine serum albumin (BSA) (cat. no. 0218054950) was purchased from MP Biomedicals (CA, USA). Enhanced chemiluminescence (ECL) reagent (cat. no. 1705062) was purchased from Bio–Rad (CA, USA). TRIzol reagent (cat. no. 15596018), first strand cDNA synthesis kit (cat. no. K1612), and PowerUp™ SYBR® Green Master Mix (cat. no. A25742) were purchased from Invitrogen (CA, USA). Dihydroethidium (DHE) (cat. no. FS0459) was obtained from Fushen Biotechnology (Shanghai, China). Normal goat serum (cat. no. ZLI-9056) was purchased from ZSGB-BIO (Beijing, China). Tween-20 (cat. no. 1247) and Triton X-100 (cat. no. 1139) were purchased from BioFroxx (Hessen, Germany). Antifade mounting medium (cat. no. P0126) and antifade mounting medium with 4′,6-diamidino-2-phenylindole (DAPI) (cat. no. P0131) were purchased from Beyotime (Shanghai, China).

The primary antibodies used targeted the following proteins: GAPDH (cat. no. 5174), *β*-Tubulin (cat. no. 2128), matrix metalloproteinase 2 (MMP-2, cat. no. 87809), phospho-p38 MAPK (Thr180/Tyr182, cat. no. 4511), p38 (cat. no. 8690), phospho-I*κ*B*α* (Ser32) (cat. no. 5209), I*κ*B*α* (cat. no. 4814), IKK*α* (cat. no. 2682), IKK*β* (cat. no. 2678), and phospho-IKK*α*/*β* (Ser176/180, cat. no. 2697) were purchased from Cell Signaling Technology, Inc. (Danvers, MA, USA). Collagen type I *α* 1 (Colla1, cat. no. ab34710), TGF-*β* (cat. no. ab179695), TNF alpha (TNF-*α*, cat. no. ab6671), alpha smooth muscle actin (*α*-SMA, cat. no. ab32575), and Ki67 (cat. no. ab16667) were purchased from Abcam (Cambridge, MA, USA). Antibodies targeting 4-hydroxynonenal antibody (4-HNE, cat. no. bs-6313R) were purchased from Bioss (Beijing, China). CD45 antibody (cat. no. 30-F11) was purchased from Invitrogen (Waltham, MA, USA), and IL-10 antibody (cat. no. E-AB-70235) was purchased from Elabscience (Wuhan, China). An antibody targeting *α*-smooth muscle (cat. no. C6198) was purchased from Sigma–Aldrich. The secondary antibodies used were horseradish peroxidase (HRP)-conjugated anti-rabbit and anti-mouse secondary antibodies (cat. no. 7074 and 7076), and they were purchased from Cell Signaling Technology, Inc. Alexa Fluor 488 goat anti-rabbit (111-545-003), Cy3 goat anti-rabbit (cat. no. 111-165-003), and Cy3 goat anti-rat (cat. no. 112-165-167) antibodies were obtained from Jackson ImmunoResearch (West Grove, PA, USA).

### 2.2. Establishment of an Ang II-Induced Hypertensive Mouse Model

All experiments involving animals were in agreement with existing guidelines for the Care and Use of Laboratory Animals published by the National Institutes of Health (publication No. 85-23, revised 1996) and were authorized by The Institutional Animal Care and Use Committee (IACUC) of Nanjing Medical University. The ethical approval numbers are IACUC-1701020 and IACUC-14030149.

Genetically consistent littermates of C57BL/6J mice (males, 8 weeks old, 23–26 g, *n* =8–10) were subjected to either Ang II (1.44 mg/kg/day) or saline administered by subcutaneous osmotic minipumps for 2 weeks. Then, these mice received subcutaneous injection of mouse recombinant IL-10 (50 *μ*g/kg b.w.) or saline on days 0, 1, 3, 5, 7, 9, 11, and 13 [14]. Mice were assigned to the following groups at random: saline group, Ang II group, IL-10 group, and Ang II + IL-10 group. Systolic blood pressure (SBP), diastolic blood pressure (DBP), and mean arterial blood pressure (MAP) were measured in a conscious state with the tail-cuff system using BP2000 (Visitech Systems, Apex, NC, USA) according to the manufacturer's protocol.

### 2.3. Human Blood Samples for Enzyme-Linked Immunosorbent Assay (ELISA)

Experimental procedures using human blood samples conformed to the principles outlined in the Declaration of Helsinki and were performed with the approval of the Ethics Committee of the First Affiliated Hospital of Nanjing Medical University (No. 2019-SR-097). A total of 36 blood samples were obtained from subjects who did not have hypertension or related diseases that affect blood pressure, and 32 blood samples were collected from hypertensive patients (defined as DBP ≥90 mmHg and/or SBP ≥140 mmHg) who did not take antihypertensive medication. Venous blood was collected in vacuum containers and stored at −80°C. IL-10 was measured using an ELISA kit for human IL-10 in human plasma according to the manufacturer's protocol.

### 2.4. Isolation and Culture of Mouse Vascular Adventitial Fibroblasts (mVAFs)

Healthy 12-week-old C57BL/6J mice (Vital River Laboratory Animal Technology, Beijing, China) were chosen for separation of MAFs. The thoracic aorta was isolated and collected quickly under sterilization and then rinsed with cold HBSS without CaCl_2_ or MgCl_2_. The aorta was cut open, and the inner and intermediate membranes were gently removed, leaving only the outer membrane. The membrane was minced into small pieces carefully (1 mm^3^), soaked in serum-free DMEM with type II collagenase (2 mg/mL) for approximately 1 h, and placed in a constant temperature shaker at 37°C. The supernatant was collected every 0.5 h and rotated at 1000 rpm for 5 min. The pellet was resuspended in DMEM supplemented with 15% (v/v) FBS and 1% (v/v) penicillin–streptomycin, and the cells were cultured in a humidified incubator with 5% CO_2_ at 37°C. The medium was changed every 2 days, and cell passage was carried out with EDTA-free trypsin at 80–90% cell confluence. Cell passages 3-6 were used for the following experiments.

### 2.5. Angiotensin II Treatment In Vitro

When the primary mVAFs reached 80%–90% confluency, the medium was replaced with serum-free DMEM to starve the cells 6-8 h. Then, the cells were divided into four groups: control (no treatment), Ang II (1 *μ*mol/L), IL-10 (10 ng/ml), and Ang II + IL-10 and underwent treatment for 18 hours. All reagents listed above were diluted in DMEM. Cells were then collected for subsequent experiments.

### 2.6. Quantification of Vascular Remodelling

All mice were sacrificed after Ang II or saline treatment for 14 days. For histological analysis, we separated mouse thoracic aortas, which were placed in 10% formalin and embedded in paraffin. Serial aortic sections were located between 5 mm and 10 mm posterior to the left subclavian artery and stained with haematoxylin and eosin (H&E) and Masson's trichrome. The aortic media area, media thickness/lumen diameter, and fibrosis area (blue indicates collagen deposition, with a mean of six sections of at least five mice per group) were quantified using Image-Pro Plus software (Media Cybernetics, Inc., Silver Spring, MD, USA).

### 2.7. Quantitative PCR (qPCR)

TRIzol was used to extract total RNA from the aortic tissue or lysed mVAFs. After quantifying the concentration of total mRNA in each sample, 1 *μ*g of the RNA was reverse transcribed into cDNA with a Transcriptor First Strand cDNA Synthesis Kit (cat. no. K1612; Invitrogen; Thermo Fisher Scientific, Inc.). qPCR analysis (ABI-Prism 7900 sequence detection system) was performed on cDNA with PowerUp™ SYBR® Green Master Mix. Each sample was analyzed in triplicate, and the relative mRNA expression levels were calculated by the 2^-*ΔΔ*Cq^ method. Target genes were normalized to endogenous glyceraldehyde 3-phosphate dehydrogenase (GAPDH). Primers are shown in Supplementary Table 1 (Table S1). Fold differences were then calculated for each treatment group using CT values normalized to the control values.

### 2.8. Western Blotting

The expression levels of phosphorylated and total protein in the aortic tissue of mice or lysed mVAFS were analyzed by western blotting. Proteins were separated by 10~15% SDS–polyacrylamide gel electrophoresis and transferred onto a 0.2 *μ*m pore size polyvinylidene difluoride membrane. To reduce nonspecific binding proteins, the membranes were incubated with Tris-buffered saline containing 1% Tween-20 (TBST) and 5% bovine serum albumin as blocking buffer for two hours at room temperature. Incubation with primary antibody was performed overnight at 4°C in blocking solution. Then, the membranes were incubated with HRP-conjugated secondary antibody in blocking buffer for 1 hour at room temperature. Membranes were then washed three times for 15 minutes each in TBST on a shaker. The blots were detected with ECL reagent and exposed on a ChemiDoc MP imager (Bio–Rad Laboratories). Image Lab™ (Bio–Rad Laboratories) software was used to analyze the protein levels. The primary antibodies used were as follows: anti-collagen type I *α* 1 (1 : 1000), anti-TGF-*β* (1 : 1000), anti-MMP2 (1 : 1000), anti-TNF-*α* (1 : 1000), anti-PCNA (1 : 1000), anti-p-p38 (1 : 1000), anti-p38 (1 : 1000), anti-phospho-I*κ*B*α* (Ser32) (1 : 1000), anti-I*κ*B*α* (1 : 1000), anti-IKK*α* (1 : 1000), anti-IKK*β* (1 : 1000), anti-phospho-IKK*α*/*β* (Ser176/180) (1 : 1000), anti-GAPDH (1 : 1000), and anti-*β*-Tubulin (1 : 1000). The following secondary antibodies were used: HRP-conjugated anti-rabbit and anti-mouse antibodies (1 : 5000).

### 2.9. Immunofluorescence

Mice were anaesthetized and perfused with 20 ml ice-cold PBS via the left ventricle. Thoracic aortas were harvested for staining, and connective and perivascular adipose tissues were removed under a stereo zoom microscope (SMZ-168, Motic) in dissection dishes using micro scissors and micro forceps. Tissue samples were fixed with 10% neutral formalin overnight at 4°C and embedded in paraffin. Tissue sections (4 *μ*m) located between 5 mm and 10 mm posterior to the left subclavian artery were dewaxed in water. VAFs were washed with PBS and fixed with 4% paraformaldehyde for 10 minutes. Then, tissue sections and VAFs were permeabilized for 10 minutes using 0.5% Triton X-100. To decrease nonspecific binding levels, sections were incubated with 5% BSA plus 10% goat serum and 0.05% Triton X-100 in PBS as blocking buffer for one hour at room temperature. Subsequently, samples were stained with primary antibodies at 4°C overnight and washed three times for two minutes each with PBS. Then, sections were incubated with secondary antibody for 1 hour at room temperature followed by three two-minute washes with PBS. The final wash was performed with ultrapure water, after which samples were mounted between microscope slides and coverslips (10212450C, CITOTEST Scientific, China) with antifade mounting medium with DAPI. Fluorescent images were captured with identical settings on a Carl Zeiss Axioskop microscope (Carl Zeiss AG, Oberkochen, Germany) with a minimum of 5 randomly selected fields. Images were further analyzed by ImageJ software (version 1.8.0; National Institutes of Health, Bethesda, MD, USA) in a double-blinded manner. The primary antibodies used were anti-TNF-*α* (1 : 200, Abcam), anti-4-HNE (1 : 200, Bioss), anti-IL-10 (1 : 200, Elabscience), anti-CD45 (1 : 100, Invitrogen), anti-Ki67 (1 : 100, Abcam), anti-*α*-SMA (1 : 200, Abcam), and Cy3-conjugated anti-*α*-SMA (1 : 1000, Sigma). The secondary antibodies used were Cy3 goat anti-rabbit, Cy3 goat anti-rat, and Alexa Fluor 488 goat anti-rabbit (1 : 400, Jackson ImmunoResearch Inc.).

### 2.10. Dihydroethidium Staining

Mice were sacrificed after anaesthetization, and the thoracic aortas were dissected in PBS before embedding in O.C.T. compound (4583, Sakura Finetek, USA). The embedded samples were immediately placed at -80°C. To investigate the level of vascular superoxide anions, 4 *μ*m sections were mounted on adhesion microscope slides (188105, CITOTEST Scientific, China) and incubated with 1 *μ*mol/L DHE in PBS for 30 minutes at room temperature in a moisture chamber. Slides were washed two times for 3 minutes with PBS and mounted between microscope slides and coverslips with antifade mounting medium. Samples were observed and imaged on a Carl Zeiss Axioskop microscope. The laser settings were unchanged for all sections in each group to enable direct comparisons. ImageJ software was applied to analyze the fluorescence intensity.

### 2.11. Statistical Analysis

All measurement data are presented as the mean ± standard error of the mean (SEM) from three independent experiments. GraphPad Prism 8.0 (GraphPad Software) was used to compare treatment group values and the appropriate control group values. Data analyses for normality and equal variance with the Shapiro–Wilk test (*p* < 0.05) and the F test (*p* > 0.05) were the first steps. For data with a normal distribution, we compared 2 groups via 2-tailed Student's *t* test and multiple groups via one-way analysis of variance (ANOVA) with the Bonferroni post hoc test. In turn, for nonnormally distributed data, the Mann–Whitney U test was used to compare 2 groups. *p* < 0.05 was considered statistically significant.

## 3. Results

### 3.1. IL-10 Does Not Alter Blood Pressure in Ang II-Induced Hypertension

**Figure 1:**
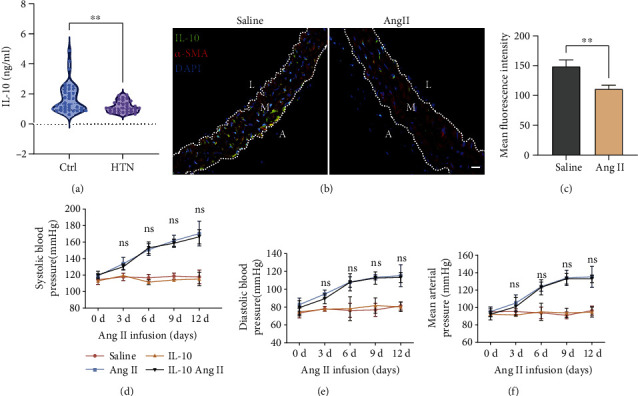
IL-10 decreased in hypertensive subjects and had no influence on blood pressure in Ang II-induced hypertension. (a) Plasma from normotensive subjects (Ctrl, *n* =36) and patients with hypertension (HTN, *n* =32) was collected to measure IL-10 by ELISA, ∗∗*p* < 0.01. (b) and (c) Representative images and dual immunofluorescence staining of IL-10 (green) and *α*-SMA (red) in aortas from saline- and Ang II-treated mice (*n* =7 for each group, scale bar, 20 *μ*m) and quantification of the ratio of the IL-10-positive area to the *α*-SMA-positive area. Error bars show the S.E, ∗∗*p* < 0.01. (d)—(f) Blood pressure measurements of SBP, DBP, and MAP in IL-10 group and control group mice infused with Ang II or saline (*n* =8-10 for each group, ns, no sense, IL-10 Ang II vs. Ang II). Error bars show the S.D.

To explore whether IL-10 is involved in hypertension-related vascular remodelling, we first measured the plasma IL-10 levels in normal blood pressure subjects and patients with untreated hypertension. Circulating IL-10 levels were significantly decreased in essential hypertensive patients compared to normotensive subjects (Figure 1(a)). The primary aim of this research was to explore whether treatment with recombinant IL-10 affects both blood pressure and vascular remodelling in hypertension caused by Ang II. We further detected the expression of IL-10 in the blood vessels of mice with 2 weeks of Ang II treatment at 1.44 mg/kg per day. The immunofluorescence staining results showed that IL-10 was significantly decreased in aortic vessel walls after Ang II treatment (Figures 1(b) and 1(c)). Therefore, we compared the effects of mouse IL-10 treatment at 50 *μ*g/kg/day, which was previously shown to attenuate left ventricular remodelling [14]. SBP, DBP, and MAP were assessed during 2 weeks of Ang II treatment at 1.44 mg/kg per day and IL-10 treatment. However, no significant differences were observed in the SBP, DBP, or MAP results of mice in the vehicle and IL-10 groups over time (Figures 1(d)–1(f)).

### 3.2. IL-10 Suppresses Ang II-Induced Vascular Fibrosis and Remodelling in Mice

**Figure 2:**
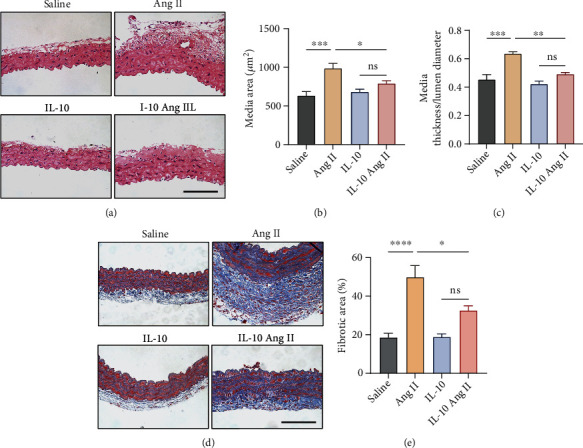
IL-10 suppresses Ang II-induced vascular fibrosis and remodelling in mice. (a)–(c) Representative images of haematoxylin and eosin (H&E) staining and quantification of vascular medial thickness and medial thickness/lumen diameter in aortas from IL-10 group and control group mice infused with Ang II or saline (scale bar, 250 *μ*m, *n* =8-10, ∗*p* < 0.01, ∗∗*p* < 0.01, ∗∗∗*p* < 0.001). Error bars show S.E. (d) and (e) Representative images of Masson staining and quantification of the fibrotic areas in aortas from IL-10 group and control group mice infused with Ang II or saline (scale bar, 100 *μ*m, *n* =8-10, ∗∗∗*p* < 0.001). Error bars show S.E.

To examine the influence of IL-10 administration on vascular remodelling, we conducted aortic morphometric analyses. Arterial wall thickness was increased in Ang II-stimulated hypertensive mice, as shown by major increases in the aortic cross-sectional area (CSA) and media-to-lumen (M/L) ratio compared to sham-operated mice. IL-10 therapy blunted hypertension-induced vascular hypertrophy independent of blood pressure (Figures 2(a)–2(c)). Masson's trichrome staining of aortic sections revealed that perivascular fibrosis was induced by Ang II infusion in saline-treated mice but not in IL-10-treated mice (Figures 2(d) and 2(e)).

**Figure 3:**
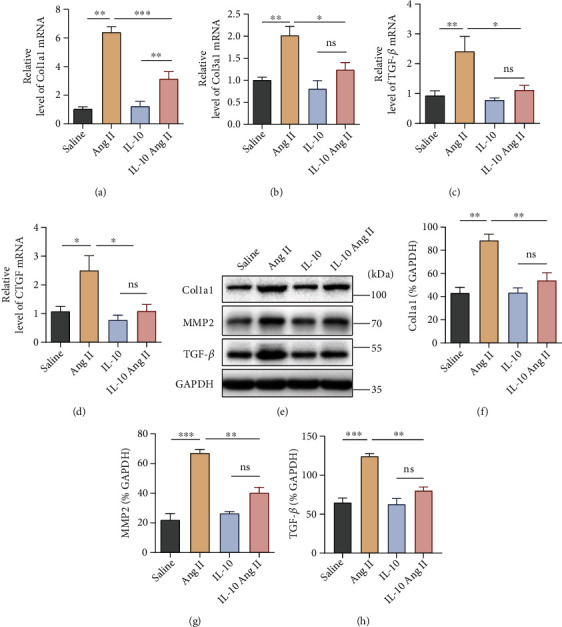
Effect of IL-10 on the expression of vascular fibrosis-associated genes. (a)–(d) The mRNA levels of fibrotic markers in the aorta, including Col1a1, CTGF, TGF-*β*, and Col3a1, were assessed by qPCR (*n* =6 for each group). (e)–(h) The protein expression levels of fibrotic markers in aortas from IL-10 group and control group mice infused with Ang II or saline were examined by western blotting. Relative protein levels were normalized to GAPDH (*n* = 3 for each group). (a)–(h) The data are representative of three independent experiments. Error bars show S.E. ∗*p* < 0.05, ∗∗*p* < 0.01, ∗∗∗*p* < 0.001.

Moreover, in vivo treatment with recombinant IL-10 induced significant decreases in Col1a1, Col3a1, TGF-*β*, and CTGF mRNA expression levels in the vessel wall compared with vehicle-treated animals after Ang II treatment (Figures 3(a)–3(d)). In addition to mRNA levels, we assessed the protein levels of fibrosis markers, including Col1a1, MMP-2, and TGF-*β*, in the IL-10 and vehicle groups after Ang II treatment. Fibrosis markers were significantly suppressed in the IL-10-treated group compared to the vehicle-treated group after Ang II treatment (Figures 3(e)–3(h)).

### 3.3. IL-10 Protected against Ang II-Induced Inflammation

**Figure 4:**
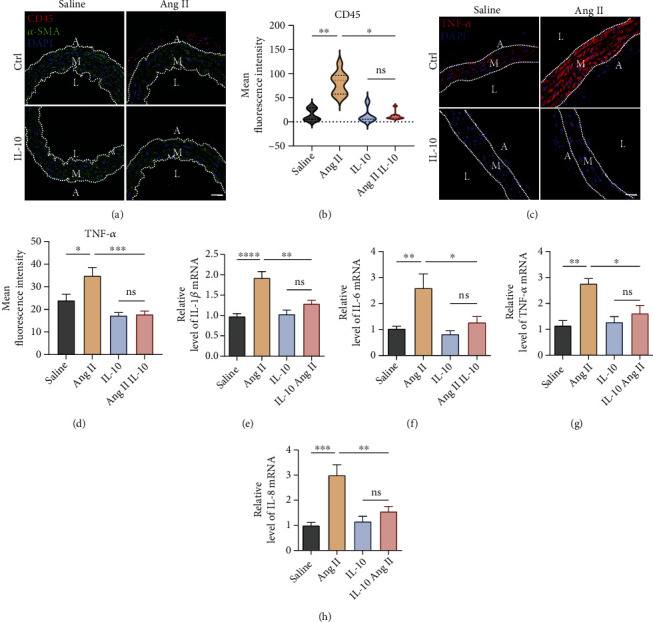
IL-10 decreased Ang II-induced vascular inflammation. (a) and (b) Representative images and quantification of vascular CD45 immunofluorescence analysis with fluorescence microscopy (*n* = 5 for each group; scale bars, 40 *μ*m). (c)–(d) Representative images and quantification of vascular TNF-*α* immunofluorescence analysis with fluorescence microscopy (*n* =6 for each group; scale bars, 40 *μ*m). (e)–(h) The mRNA levels of inflammatory markers (IL-1*β*, IL-6, IL-8, and TNF-*α*) in the aorta were determined by qPCR (*n* =6 for each group). The data are representative of three independent experiments. Error bars show S.E. ∗*p* < 0.05, ∗∗*p* < 0.01, ∗∗∗*p* < 0.001. Ctrl: control; DAPI: 4′:6-diamidino-2-phenylindole; A: adventitia; M: media; L: lumen.

Inflammation is regarded as one of the important common denominators of cardiovascular risk and arterial stiffness, and immune cell infiltration is pivotal for vascular fibrosis [15]. Immunohistochemical staining of CD45-positive cells (CD45^+^ cells) on vascular tissue sections was carried out to study inflammatory cell infiltration after 14 days of Ang II treatment. Infiltration of CD45^+^ cells was observed in the perivascular region after Ang II treatment, and IL-10 treatment significantly inhibited this infiltration (Figures 4(a) and 4(b)). To investigate the effect of IL-10 on inflammatory responses, we analyzed the immunofluorescence staining of proinflammatory cytokine levels of TNF-*α* in vascular tissue sections. IL-10 treatment significantly inhibited the expression of TNF-*α* on aortic walls (Figures 4(c) and 4(d)). Furthermore, we detected the proinflammatory cytokine levels of IL-1*β*, IL-6, IL-8, and TNF-*α* after Ang II treatment by qPCR. The mRNA expression levels of proinflammatory cytokines were significantly reduced in the vascular tissue of Ang II-infused and IL-10-treated mice (Figures 4(e)–4(h)).

### 3.4. IL-10 Reduced Ang II-Stimulated Vascular Oxidative Stress and Lipid Peroxidation

**Figure 5:**
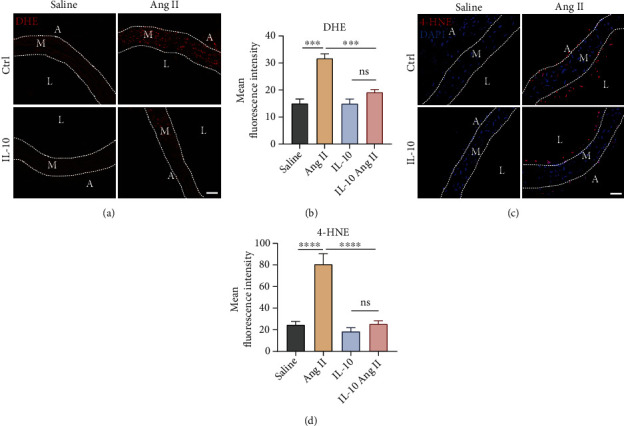
IL-10 decreased Ang II-induced vascular inflammation and lipid peroxidation. (a) and (b) Representative images and quantification of vascular DHE immunofluorescence analysis with fluorescence microscopy (*n* =6 for each group; scale bars, 40 *μ*m). (c) and (d) Representative images and quantification of vascular 4-HNE immunofluorescence analysis with fluorescence microscopy (*n* =6 for each group; scale bars, 40 *μ*m). The data are representative of three independent experiments. Error bars show S.E. ∗∗∗*p* < 0.001, ∗∗∗∗*p* < 0.0001. Ctrl: control; DHE: dihydroethidium; 4-HNE: 4-hydroxynonenal; DAPI: 4′6-diamidino-2-phenylindole; A: adventitia; M: media; L: lumen.

Given that chronic severe vascular oxidative stress can cause inflammation, hypertension, and vascular remodelling [16], we assessed the level of superoxide anion in cryosections of the aorta by dihydroethidium (DHE) staining, as shown in representative fluorescence microscopic images. The DHE signal was significantly increased in the Ang II-induced group, and treatment with IL-10 almost fully reversed this effect after Ang II treatment (Figures 5(a) and 5(b)). Lipid peroxidation produced by excessive accumulation of reactive oxygen species plays a relevant role in the onset of cardiovascular disease [17]. To evaluate the degree of lipid peroxidation, 4-HNE immunofluorescence analysis was performed. The results showed that IL-10 significantly reduced Ang II-induced lipid peroxidation in vascular walls (Figures 5(c) and 5(d)). Collectively, these results reveal that IL-10 protects Ang II-stimulated vessels with strong antioxidant effects.

### 3.5. IL-10 Inhibits Ang II-Induced Cell Proliferation, Inflammatory Responses, and Fibrosis of Mouse Vascular Adventitial Fibroblasts (mVAFs)

**Figure 6:**
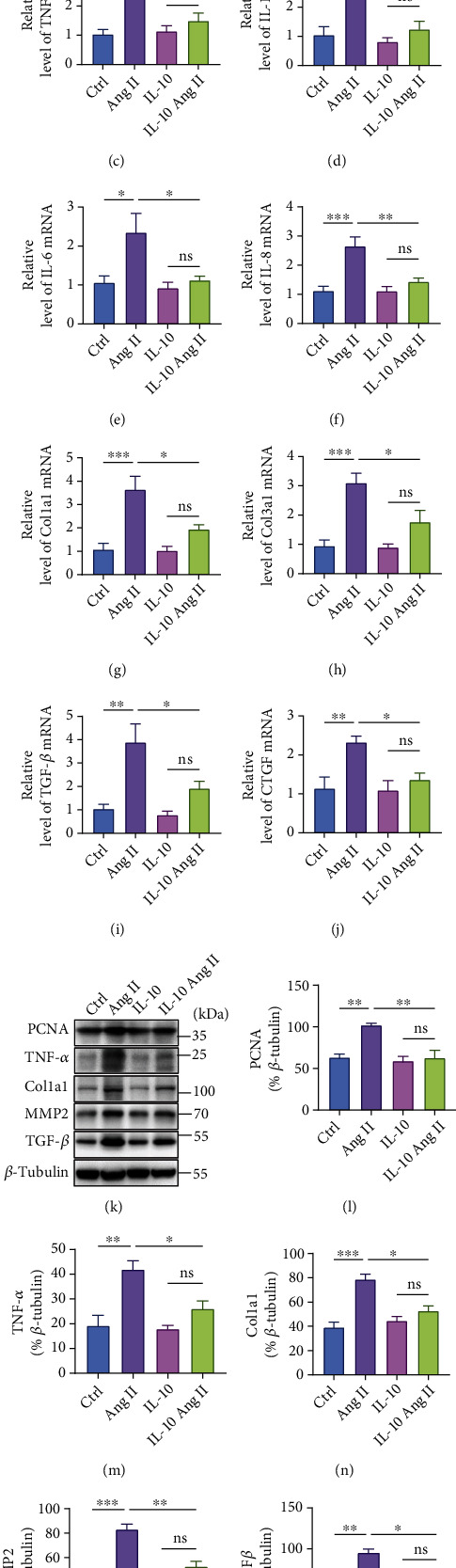
IL-10 suppresses Ang II-induced proliferation, fibrosis, and inflammation of mouse vascular adventitial fibroblasts (mVAFs). (a) and (b) Representative images of immunofluorescence colocalization staining of Ki67 (green) and DAPI (navy) in mVAFs treated with Ang II (*n* =8 for each group; scale bars, 100 *μ*m). (c)–(j) The mRNA levels of inflammatory markers (TNF-*α*, IL-1*β*, IL-6, and IL-8) and fibrotic markers (Col1*α*1, Col3*α*1, TGF-*β*, and CTGF) in mVAFs treated with Ang II were assessed by real-time PCR. (k)–(p) The protein expression levels of PCNA, TNF-*α*, and fibrotic markers were examined by western blotting in mVAFs treated with Ang II. Relative protein levels were normalized to GAPDH. The data are representative of three independent experiments. Error bars show S.E. ∗*p* < 0.05, ∗∗*p* < 0.01, ∗∗∗*p* < 0.001.

To further explore the role of IL-10 in the protective effect against Ang II-induced proliferation and fibrosis of the adventitia, we isolated and cultured mVAFs. Ki67 immunofluorescence and the protein level of PCNA in mVAFs were quantified to verify the effect of IL-10 on cell proliferation. Our results showed that Ang II significantly increased the number of Ki67-positive cells and the expression of PCNA in mVAFs, while IL-10 significantly inhibited the increase in Ki67-positive cells and downregulated the expression of PCNA after Ang II treatment; this indicates that IL-10 inhibited the proliferation of Ang II-induced mVAFs (Figures 6(a), 6(b), 6(k), and 6(l)). In addition, we detected the proinflammatory cytokine levels of TNF-*α*, IL-1*β*, IL-6, and IL-8 after Ang II treatment by qPCR. The mRNA expression levels of proinflammatory cytokines revealed significant declines in Ang II-induced mVAFs after IL-10 treatment (Figures 6(c)–6(f)). The protein level of TNF-*α* exhibited a similar change relative to qPCR (Figures 6(k) and 6(m)). Moreover, in vitro treatment with recombinant IL-10 induced significant decreases in Col1a1, Col3a1, TGF-*β*, and CTGF mRNA expression in mVAFs compared with vehicle-treated mVAFs after Ang II treatment (Figures 6(g)–6(j)). Beyond the mRNA levels, similar changes in fibrosis markers, including Col1a1, MMP2, and TGF-*β*, were observed at the protein level in mVAFs (Figures 6(k) and 6(n)–(p)). Consistent with the in vivo results, these findings indicated that IL-10 treatment significantly inhibited Ang II-induced proliferation, inflammatory responses, and fibrosis in mVAFs in vitro.

### 3.6. IL-10 Suppresses the Phosphorylation of p38 MAP Kinase and NF-*κ*B in Ang II-Induced Vascular Fibrosis

**Figure 7:**
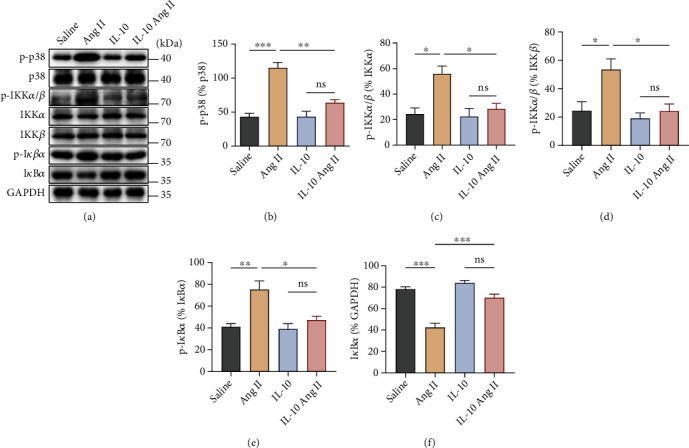
IL-10 suppresses the phosphorylation of p38 MAP kinase and NF-*κ*B in Ang II-induced vascular fibrosis. (a)–(f) The protein expression levels of p38 MAP kinase and the NF-*κ*B pathway in aortas from IL-10 group and control group mice infused with Ang II or saline were examined by western blotting (*n* = 3 for each group). The data are representative of three independent experiments. Error bars show S.E. ∗*p* < 0.05, ∗∗*p* < 0.01, ∗∗∗*p* < 0.001.

**Figure 8:**
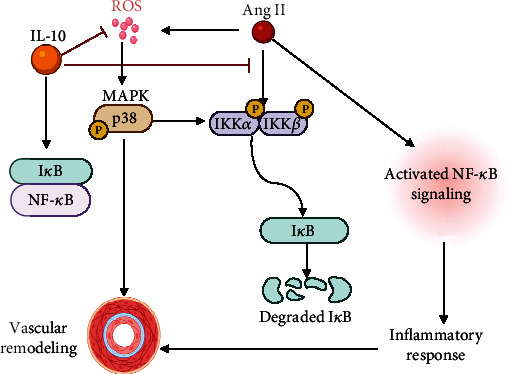
Schematic illustration of IL-10 attenuating Ang II-induced aortic remodelling by inhibiting oxidative stress-induced activation of the vascular p38 and NF-*κ*B pathways. I*κ*B binds to NF-*κ*B to inhibit activation of NF-*κ*B signalling. Ang II increases ROS production and phosphorylation of IKK, leading to further phosphorylation and degradation of I*κ*B, thereby activating the NF-*κ*B pathway and resulting in the release of inflammatory factors and vascular remodelling. IL-10 inhibits the activation of NF-*κ*B signalling by inhibiting ROS and the phosphorylation of p38 and IKK, thus inhibiting the excessive secretion of inflammatory factors and attenuating vascular remodelling. ROS: reactive oxygen species: IKK: I*κ*B kinase.

To investigate the influence of IL-10 treatment on the expression of intracellular signalling pathways, we detected the protein levels of phosphorylated p38 MAPKs after 14 days of Ang II treatment. Western blot analysis showed that phosphorylation of p38 MAP kinase (p-p38) was augmented in the vascular tissue after Ang II treatment. However, IL-10 treatment significantly suppressed p-p38 expression (Figures 7(a) and 7(b)). The transcription factor NF-*κ*B is a transcriptional activator of inflammatory mediators, such as cytokines. Western blot assays showed that IKK phosphorylation increased with Ang II treatment, but IKK phosphorylation was attenuated by administration of IL-10 (Figures 7(a), 7(c), and 7(d)). Activation of NF-*κ*B is triggered by signal-induced degradation of the I*κ*B-*α* protein. Furthermore, Ang II stimulation decreased the expression level of I*κ*B*α*, an NF-*κ*B inhibitor, but IL-10 treatment reversed this decline (Figures 7(a) and 7(e)). These results provide evidence that IL-10 suppresses the phosphorylation of p38 MAP kinase and the NF-*κ*B signalling pathway (Figure 8).

## 4. Discussion

In this study, we demonstrated that recombinant IL-10 reverses the structural and molecular biological alterations induced by Ang II in aortas even in the absence of any change in blood pressure. This response was related to the antioxidant and anti-inflammatory effects of IL-10 treatment, which inhibited the activation of vascular p-p38 and NF-*κ*B, thereby preventing vascular fibrosis and remodelling.

Thickening of the intima and media is a characteristic of the vascular remodelling of the large arteries caused by hypertension [18]. In the process of hypertension, the arterial wall thickens and hypertrophies, which eventually leads to increased vascular stiffness and decreased arterial compliance [19]. Consequently, increased CSA and the M/L ratio of aortas are crucial markers of vascular remodelling [20]. In this regard, a clinical study showed that enhanced aortic stiffness and decreased arterial compliance correlated significantly with negative end-organ functions in the heart and kidney in mild hypertension patients [21]. Recent studies have confirmed that hypertensive patients have a significantly increased wall-to-lumen ratio compared to normotensive controls [22, 23]. Our results showed that IL-10 treatment reversed hypertension-induced vascular hypertrophy by decreasing aortic CSA and the M/L ratio.

Studies have confirmed that the infiltration of inflammatory cells into the vascular wall can lead to vascular remodelling, which contributes to hypertension. It has become increasingly clear in recent years that hypertension is an inflammatory process that involves the transmigration and aggregation of congenital and adaptive inflammatory cells in affected arterial tissue, releasing cytokines and promoting oxidative stress [24]. The infiltration of immune cells into large conductive arteries is a significant characteristic of hypertension in animal models [25, 26]. Myriad chemokines and cytokines have been identified to regulate both inflammatory reactions and the signalling pathways that guide their synthesis. In the past few years, important roles of at least 5 cytokines have been identified in hypertension, including IL-17, interferon *γ* (IFN-*γ*), TNF-*α*, IL-6, and IL-10 [24]. IL-10 is an anti-inflammatory cytokine originally discovered based on its ability to inhibit the production of IL-2 and IFN-*γ* [27]. Didion et al. defined IL-10 as having a critical role in modulating endothelial function in hypertension [28]. CD4^+^CD25^+^ natural regulatory T cells release IL-10, which improves the function of microvascular endothelial cells in hypertensive mice by inhibiting NADPH oxidase activity [29]. Our study showed that IL-10 treatment significantly decreased TNF-*α* infiltration and the expression of IL-1*β*, IL-6, IL-8, and TNF-*α* in Ang II-induced vascular inflammation. In summary, these studies support the anti-inflammatory effects of IL-10 in hypertension and vascular remodelling.

Vascular oxidative stress injury is accompanied by many general conditions related to hypertension [16]. Reactive oxygen species (ROS) are an important mediator of hypertension caused by exposure to Ang II [30] and are involved in angiotensin II-induced vascular injury [31]. Meanwhile, vascular remodelling caused by angiotensin II showed a marked increase in reactive oxygen species [32]. Seon Hwa Lee et al. found that angiotensin II could cause lipid peroxidation in human endothelial cells [33]. Yasumasa Ikeda et al. reported that lipid peroxidation and superoxide anions were increased in the mouse coronary artery and aorta by angiotensin II stimulation [34]. Eva Latorre et al. found that IL-10 counteracts proinflammatory mediator-evoked oxidative stress in the intestine and restores the altered redox equilibrium in intestinal epithelial cells [35]. In agreement with previous results, our group found that treatment with IL-10 reversed Ang II induction of vascular oxidative stress and lipid peroxidation; thus, IL-10 showed a strong antioxidant effect.

IL-10 is a pleiotropic cytokine that affects cells through a wide range of signal cascades in a gene-dependent manner. The roles of p38-dependent and p38-independent NF-*κ*B activation in the progression of inflammation and vascular fibrosis are well established [36, 37]. The mitogen-activated protein kinase (MAPK) family may be a potential target for reactive oxygen species [38]. p38 MAPK can be strongly activated by H_2_O_2_ produced in cells induced by agonists or exogenous reactive oxygen species. In fact, when the increase in intracellular H_2_O_2_ production induced by Ang II was blocked, the activation of p38 MAPK was significantly inhibited [38]. Furthermore, activation of p38 MAPK plays a vital role in Ang II-dependent vascular injury [39]. We found that IL-10 treatment strongly suppressed p38 phosphorylation in Ang II-induced vascular remodelling. p38 MAPK is known as a highly redox-sensitive MAPK, and Ang II-induced activation of p38 MAPK is mediated by intracellular reactive oxygen species generation. This pathway may be the link between hormone stimulation to produce reactive oxygen species and vascular remodelling.

We have demonstrated that IL-10 exerts its anti-inflammatory effects by inhibiting the activation of the NF-*κ*B pathway. NF-*κ*B regulates the expression of related genes and plays an important role in vascular remodelling by affecting the balance of pro- and anti-inflammatory factors. A combination of chemokines and cytokines, such as TNF-*α* and IL-1*β*, are regulated by the NF-*κ*B pathway [40]. NF-*κ*B plays a critical role in Ang II-induced vascular inflammation and is crucial for macrophage recruitment at the interstitium of affected arteries [41]. Treatment with IL-10 can both suppress IKK phosphorylation and protect against I*κ*B*α* degradation in Ang II-induced vascular remodelling, suggesting that the effect of IL-10 on the inflammatory response is associated with the inhibition of the NF-*κ*B pathway.

In conclusion, we demonstrated that IL-10 treatment reverses vascular structural alterations in hypertension without normalizing blood pressure. This response is probably mediated by the antioxidant and anti-inflammatory effects of IL-10, which blunt oxidative stress-induced activation of vascular p38 and the NF-*κ*B pathway, alleviating vascular remodelling. Whether IL-10 interferes with these pathophysiological mechanisms may become a therapeutic modality for other cardiovascular diseases in the future, and this requires further research.

## Data Availability

The authors confirm that the data supporting the findings of this study are available within the article and its supplementary materials.

## Abbreviations

IL-10: Interleukin 10
Ang II: Angiotensin II
MAPK: Mitogen-activated protein kinase
FBS: Foetal bovine serum
CTGF: Connective tissue growth factor
MMP: Matrix metalloproteinase
mVAFs: Mouse vascular adventitial fibroblasts
TNF-*α*: Tumour necrosis factor-*α*
NF-*κ*B: Nuclear factor-*κ*B
ECM: Extracellular matrix
STAT-3: Signal transducer and activators of transcription 3.

## Supplementary Materials

Supplementary Materials Supplementary Figure 1. Uncropped western blots (related to Figure 3). The blots were developed with enhanced chemiluminescence reagent and exposed on a ChemiDoc MP imager (Bio-Rad). Supplementary Figure 2. Uncropped western blots (related to Figure 6). The blots were developed with enhanced chemiluminescence reagent and exposed on a ChemiDoc MP imager (Bio-Rad). Supplementary Figure 3. Uncropped western blots (related to Figure 7). The blots were developed with enhanced chemiluminescence reagent and exposed on a ChemiDoc MP imager (Bio-Rad). Supplementary Table 1. Primers used for quantitative polymerase chain reaction, Related to Methods.
