# Supplementary material for: Interleukin 10 Attenuates Angiotensin II-Induced Aortic Remodelling by Inhibiting Oxidative Stress-Induced Activation of the Vascular p38 and NF-κB Pathways
Source: Oxid Med Cell Longev. 2022 Apr 26;2022:8244497. doi: 10.1155/2022/8244497 (PMC9072025; doi:10.1155/2022/8244497)
Supplement: Supplementary Materials — Supplementary Figure 1. Uncropped western blots (related to Figure 3). The blots were developed with enhanced chemiluminescence reagent and exposed on a ChemiDoc MP imager (Bio-Rad). Supplementary Figure 2. Uncropped western blots (related to Figure 6). The blots were developed with enhanced chemiluminescence reagent and exposed on a ChemiDoc MP imager (Bio-Rad). Supplementary Figure 3. Uncropped western blots (related to Figure 7). The blots were developed with enhanced chemiluminescence reagent and exposed on a ChemiDoc MP imager (Bio-Rad). Supplementary Table 1. Primers used for quantitative polymerase chain reaction, Related to Methods. [file 8244497.f1.docx]

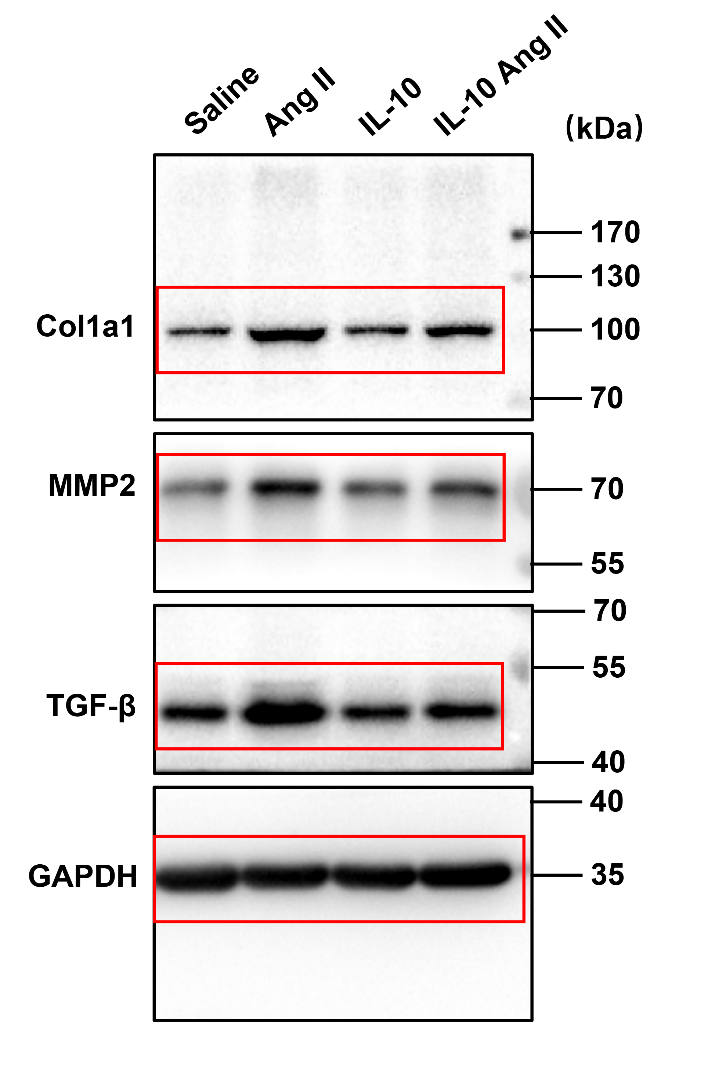


**Supplementary Figure 1.** **Uncropped Western blots (Related to Figure 3).**

The blots were developed with enhanced chemiluminescence reagent and exposed on a ChemiDoc MP imager (Bio-Rad).


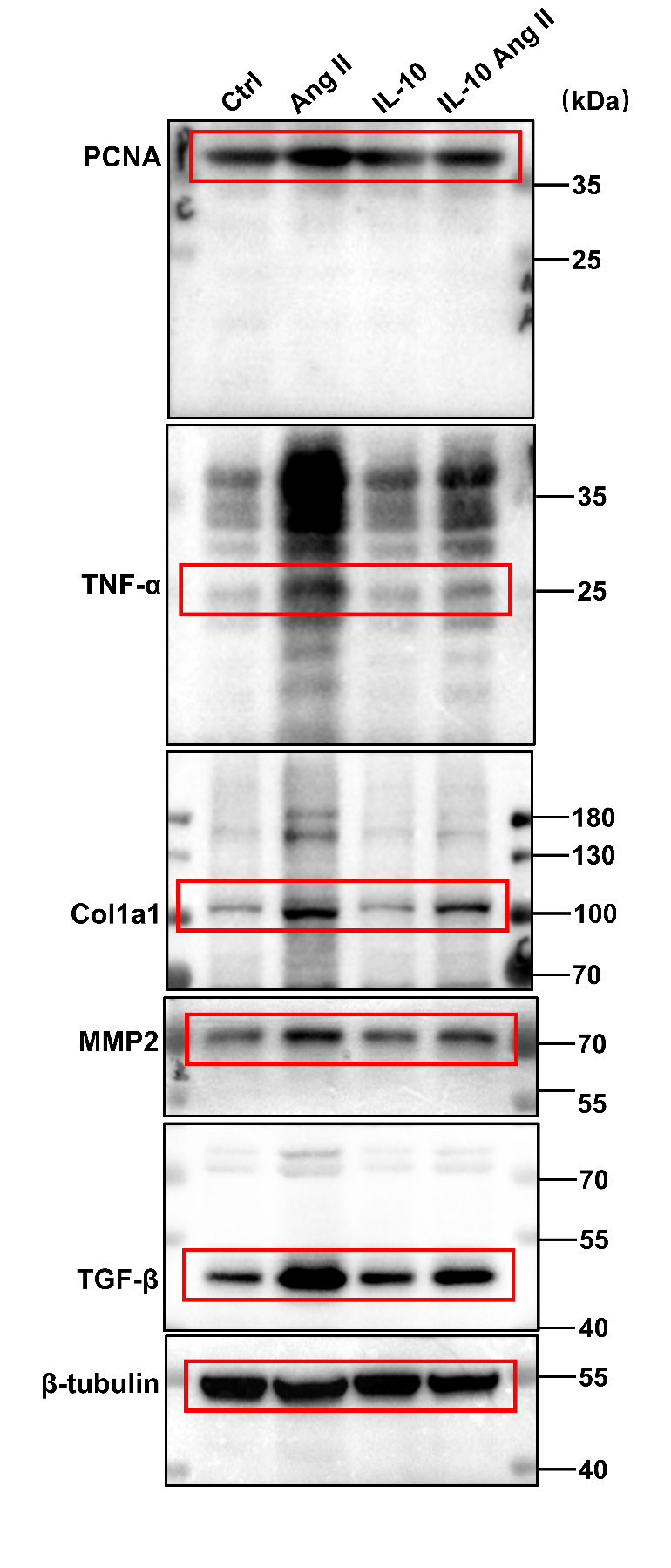


**Supplementary Figure 2.** **Uncropped Western blots (Related to Figure 6).**

The blots were developed with enhanced chemiluminescence reagent and exposed on a ChemiDoc MP imager (Bio-Rad).


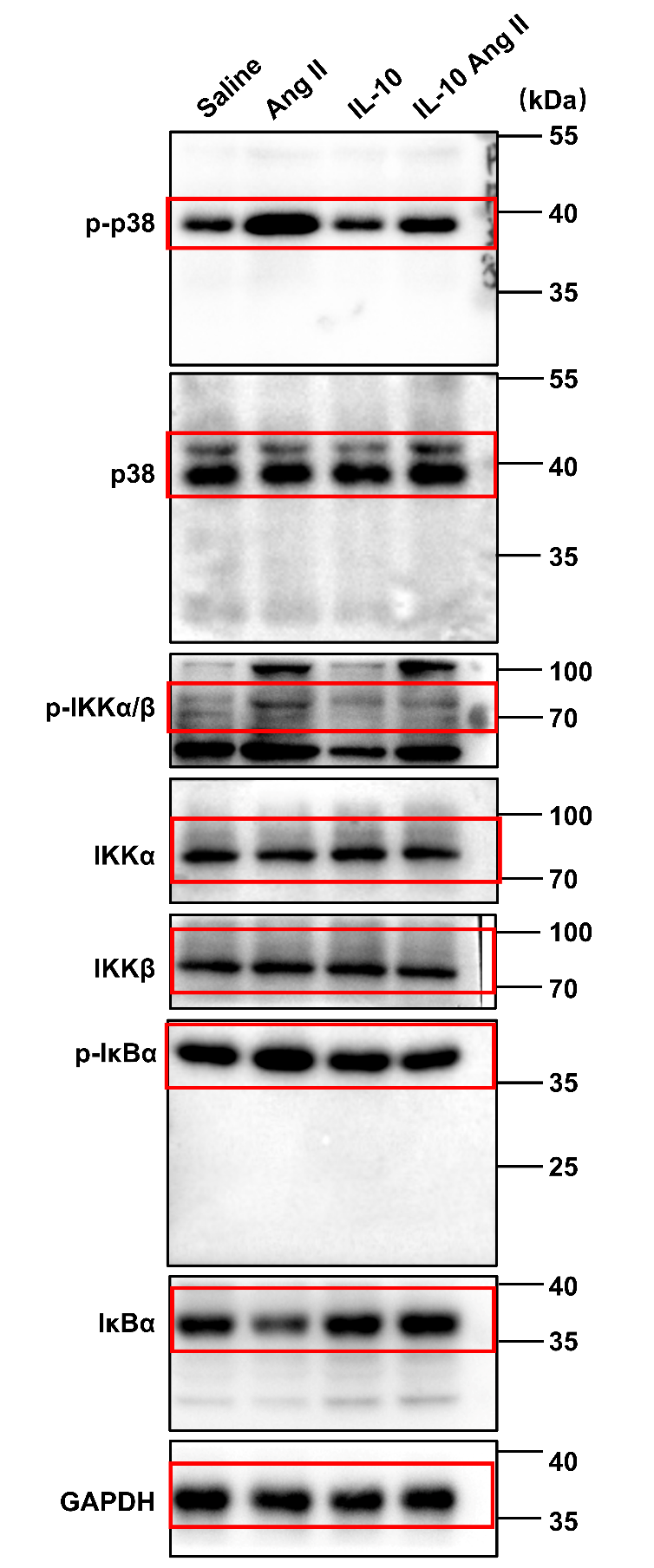


**Supplementary Figure 3.** **Uncropped Western blots (Related to Figure 7).**

The blots were developed with enhanced chemiluminescence reagent and exposed on a ChemiDoc MP imager (Bio-Rad).

**Supplementary Table 1. Primers used for quantitative polymerase chain reaction, Related to Methods.**

| Primers |  | Sequence (5'--3') | Species |
| --- | --- | --- | --- |
| Col1a1 | Forward | GCTCCTCTTAGGGGCCACT | Mouse |
|  | Reverse | CCACGTCTCACCATTGGGG |  |
| CTGF | Forward | GGGCCTCTTCTGCGATTTC | Mouse |
|  | Reverse | ATCCAGGCAAGTGCATTGGTA |  |
| TGF-β | Forward | CTCCCGTGGCTTCTAGTGC | Mouse |
|  | Reverse | GCCTTAGTTTGGACAGGATCTG |  |
| Col3a1 | Forward | AGAACCTGGCCGAGATG | Mouse |
|  | Reverse | TGGACTTCCGGGCATAC |  |
| GAPDH | Forward | AGGTCGGTGTGAACGGATTTG | Mouse |
|  | Reverse | TGTAGACCATGTAGTTGAGGTCA |  |
| IL-1β | Forward | TGGACCTTCCAGGATGAGGACA | Mouse |
|  | Reverse | GTTCATCTCGGAGCCTGTAGTG |  |
| IL-6 | Forward | TACCACTTCACAAGTCGGAGGC | Mouse |
|  | Reverse | CTGCAAGTGCATCATCGTTGTTC |  |
| IL-8 | Forward | GGTGATATTCGAGACCATTTACTG | Mouse |
|  | Reverse | GCCAACAGTAGCCTTCACCCAT |  |
| TNF-α | Forward | GGTGCCTATGTCTCAGCCTCTT | Mouse |
|  | Reverse | GCCATAGAACTGATGAGAGGGAG |  |
